# Cluster-based stability evaluation in time series data sets

**DOI:** 10.1007/s10489-022-04231-7

**Published:** 2022-12-13

**Authors:** Gerhard Klassen, Martha Tatusch, Stefan Conrad

**Affiliations:** Heinrich-Heine-University Düsseldorf, Düsseldorf, Germany

**Keywords:** Time series clustering, Over-time stability evaluation, Evolutionary clustering, Anomalous subsequences

## Abstract

In modern data analysis, time is often considered just another feature. Yet time has a special role that is regularly overlooked. Procedures are usually only designed for time-independent data and are therefore often unsuitable for the temporal aspect of the data. This is especially the case for clustering algorithms. Although there are a few evolutionary approaches for time-dependent data, the evaluation of these and therefore the selection is difficult for the user. In this paper, we present a general evaluation measure that examines clusterings with respect to their temporal stability and thus provides information about the achieved quality. For this purpose, we examine the temporal stability of time series with respect to their cluster neighbors, the temporal stability of clusters with respect to their composition, and finally conclude on the temporal stability of the entire clustering. We summarise these components in a parameter-free toolkit that we call **Cl**uster **O**ver-Time **S**tability **E**valuation (CLOSE). In addition to that we present a fuzzy variant which we call FCSETS (**F**uzzy **C**lustering **S**tability **E**valuation of **T**ime **S**eries). These toolkits enable a number of advanced applications. One of these is parameter selection for any type of clustering algorithm. We demonstrate parameter selection as an example and evaluate results of classical clustering algorithms against a well-known evolutionary clustering algorithm. We then introduce a method for outlier detection in time series data based on CLOSE. We demonstrate the practicality of our approaches on three real world data sets and one generated data set.

## Introduction

With the increase of time series (TS) data, their analysis is becoming more and more important. There are many different approaches which are all suitable for different setups. However, most of the methods target the analysis of individual time series, while only a few aim to analyse whole TS databases. Without any doubt, the information gained from a time series database can have a significant influence on the results, especially compared to an analysis applied to only one time series of the database.

A setting which illustrates this circumstance is the stock market: During an economic crisis most of the shares lose value. Regarding only one share at a time could lead to a false interpretation (e.g. an outlier sequence within the time series), while regarding all time series simultaneously the assessment would result differently.

Although the mentioned setting describes extreme circumstances, it is obvious that similar problems in analysis and interpretation also occur under normal conditions. The examination of this kind of setups can prove to be very difficult, since it can be useful not to look at the whole database at once, but to look at specific groups instead. This requires the identification of groups which is often accomplished by applying suitable clustering algorithms. Although this is a well researched topic for time independent data, approaches for time series are often insufficient, sometimes to an extent that the produced results are meaningless [25]. As this has been identified as a major problem in time series clustering, the research field *evolutionary clustering* developed. According to [8] evolutionary clustering is producing a clustering per timestamp, hence a series of clusterings. Each clustering should be similar to the clustering of its predecessor, while accurately reflecting the properties of its own data. As this definition is not regarding a certain clustering algorithm, this leads to a variety of approaches adapted to different clustering algorithms (read more about this in Section 2). There are also approaches which try to define the necessary adjustments to a standard clustering algorithm to receive an evolutionary clustering algorithm [8, 10]. However, the amount of different approaches and different clustering algorithms makes it difficult to select a suitable method for a certain task.

The detection of groups in time series can provide important insights into the data at hand. The application areas of our toolkits and the methods based on them, such as outlier detection, are diverse. One conceivable application of our methods is on medical data, where patients could be identified who were initially grouped with healthy patients and whose medical values then slowly move away from this group. Another area of application is the financial market, where, for example, companies can be grouped that behave similarly over time, so that classic company classifications such as the Standard Industrial Classification (SIC) or the North American Industry Classification System (NAICS) can be usefully supplemented. Companies that change their group more frequently in relation to other companies may have anomalies that our outlier detection method would identify. Analyses of the current Corona epidemic are also conceivable. Using data from the Coronavirus Government Response Tracker at the University of Oxford^1^, the effectiveness of government measures to contain the epidemic could be analysed. It would also be possible to identify how good the respective chosen timing of a measure was. There are countless applications where our methods can be used. In addition, all our methods are transparent and provide explainable results.

In this paper we describe two fundamental methods to evaluate time series clustering according to its over-time stability. The first algorithm CLOSE (**Cl**uster **O**ver-Time **S**tability **E**valuation) [51] is designed for multivariate time series in crisp cluster environments. Hereby we use an extended definition of evolutionary clustering: Instead of targeting the similarity of two successive clusterings we demand the similarity of a clustering to all previous clusterings. We call this the *over-time stability* and introduced it, because small changes between two timestamps could develop to huge changes over several time steps. Those changes would be overseen by considering only two consecutive timestamps. A simple example for this problem is the Covid-19 infection rate in different countries: If one country changes its cluster peers from one point in time to the other, this may be reasonable. However, if the country is changing its cluster peers in every time point, regarding solely the previous timestamp is not sufficient, since it does not hold the historical changes. Therefore this country could not be directly compared to other countries, since among those there might be countries which have changed its peers as well. Hence, the changes before the previous timestamp contribute to the overall stability.

In the course of this paper we will show that this adaptation of the definition is especially handy for certain applications like outlier detection or parameter selection for time series clustering. Our methodology is also very different from other approaches in this field of research. In contrary to a framework or an adapted clustering algorithm, CLOSE is a ready-to-use toolkit. It does not require any customization of the user-chosen clustering algorithm, instead it analyses the produced clusterings per timestamp and returns a stability score. This can be used to find the best parameter setting for the underlying clustering algorithm.

The second algorithm FCSETS (**F**uzzy **C**lustering **S**tability **E**valuation of **T**ime **S**eries) [30] is a toolkit developed for fuzzy clustering environments. It makes use of the relative assignment agreement similar to the equivalence relation in the Hüllermeier-Rifqi Index [20] and achieves a stability score by regarding the average weighted difference between the relative assignment agreements of one time series to the others. The methodology of FCSETS is very similar to the one of CLOSE and therefore further adjustments of the chosen underlying fuzzy clustering algorithm are not required. Further, we are presenting an outlier detection algorithm [50] which is an application of CLOSE. We give two variants [52] of the procedure which focus on cluster transitions and therefore are capable to detect a new sort of outliers, which are based on the *behavior* of time series in relation to its cluster peers. The implementation of the approaches as well as the generated data sets are available on Github^2^.

In order to present the results of the introduced algorithms, we use three real world data sets and one generated data set. We apply CLOSE in combination with DBSCAN [16] and K-Means [38] and FCSETS in combination with Fuzzy C-Means [6] to the selected data sets to get the best parameter settings. We qualitatively analyse the resulting clusterings and in the case of K-Means we subsequently use the possibility to compare the CLOSE score with that of the evolutionary K-Means from [8]. Further, we apply the outlier detection algorithms to the data sets and explain the results in detail.

## Related work

Since this work addresses many different problems and approaches, such as the (over-time) stability evaluation of (fuzzy) clusters and the detection of anomalous subsequences, this chapter deals with related works from various domains, as well.

### Time series clustering

There are various techniques for clustering TS data in the field of time series analysis. In [56] the approaches are divided into three categories: *raw-data-based*, *feature-based* and *model-based* clustering algorithms. The first type describes approaches, which consider the TS data without any preprocessing. The second one works with feature vectors extracted from the time series. In the third case, models are approximated for the representation of the TS data.

When considering approaches that work with the unprocessed TS data that is given, a common approach is clustering subsequences of a time series [3, 22]. As this is usually done in order to find motifs in time series, only a single TS is considered at once. This approach is controversial, since Keogh et al. state in [25] that the clustering of subsequences of a single time series is meaningless. Chen, however, argues that it is possible to obtain meaningful results if the correct distance measure is used [9]. In our context, the clustering has to be applied to multiple time series, though. Clustering subsequences has some disadvantages. First, outlier data points may have a negative impact on the results. Second, the determination of a meaningful length for the considered subsequences is difficult but needed, since the examination of subsequences of all lengths is usually very time-consuming. In our approaches, subsequences of any length can selectively be investigated and therefore provide more insights. Nevertheless, it has to be noted, that only subsequences starting at the first existing timestamp are considered. This is reasoned by the assumption, that the entire time course from the beginning is relevant for the analysis.

Another raw-data-based approach is the clustering of entire sequences [15, 17, 41]. Since potential correlations between subsequences of different TS are not recognized, this procedure is not suitable for our applications.

In our context, the exact course of time series is not relevant, but rather the trend they follow. This can e.g. be achieved by algorithms using Dynamic Time Warping (DTW) as distance measure [2, 11, 21, 33] or methods of the second type, where the sequences are transformed to feature vectors first [19]. By extracting relevant features, the exact course gets blurred. However, the problem of not recognizing correlating subsequences still persists.

When considering the third type of TS clustering, a major approach is the usage of auto-regressive moving-average models (ARIMA) [42, 57]. Therefore, an ARIMA model/mixture for every time series is fitted. Those sequences, whose models are similar to each other, are grouped to the same cluster. Also, the sequences can be modeled by the Haar Wavelet decomposition [54], their approximated seasonality [31] or with the help of Markov Chains [44]. However, all approaches share the idea of clustering whole time series. In our application, correlating subsequences and the movement of sequences with regard to their neighbors are of interest. Therefore, those methods are not applicable.

Approaches, which deal with the clustering of streaming data [18, 40] are also not comparable to our method, as they deal with other problems such as high memory requirements and time complexity, and in addition to that usually consider only one sequence at once.

### Evolutionary clustering

Evolutionary clustering describes the task of clustering temporal data per timestamp under the consideration of two criteria: on the one hand, the clustering should be reasonable for the current data, and on the other the clustering should not deviate significantly from one timestamp to another [8]. Different frameworks have been developed, which meet both criteria regarding streaming data [10], TS data [58] and dynamic networks [27]. The framework, which is presented in [8], for instance, is developed for streaming data and therefore an incremental approach, which for each timestamp *t* tries to find a clustering *C*_*t*_ that optimizes the following formula:

1 $$ sq(C_{t},M_{t}) - cp \cdot hc(C_{t-1}, C_{t})~, $$

where *sq*(*C*_*t*_,*M*_*t*_) is the *snapshot quality* regarding an object relationship matrix *M*_*t*_, *cp* is a change parameter and *hc*(*C*_*t*− 1_,*C*_*t*_) is the *history cost*. The *snapshot quality* measures the quality of a clustering at a certain time point with respect to the calculated *n* × *n* matrix *M*_*t*_ which represents the relationship of all *n* objects to each other. The history cost is calculated by the comparison of the clusterings of two consecutive time points, whereby the comparison may be applied on different data levels. For example, simply the partitions of both clusterings may be compared, or the best matching between two sets of centroids regarding KMeans [38]. The change parameter *cp* > 0 is a hyperparameter which trades off between *sq* and *hc*. With this flexible framework a stable over-time clustering may be achieved, which can be used as the underlying clustering for our outlier detection algorithm. Yet, due to the comparison of only consecutive time points, short-term changes may have a strongly negative impact on the result and large long-term changes may occur, which is not desirable.

The problem of identifying so called *Moving Clusters* [23] seems to be a closely related topic, but addresses a slightly different task. In contrast to clustering time series, this field of research deals with the detection of already given clusters that remain mostly the same with regard to their members. In addition, it is assumed that a cluster remains approximately the same size over time. This may apply to some tasks, such as herd tracking, which is examined in [23], but in most cases this requirement can not be met.

### Internal cluster evaluation measures

For the evaluation of clusters and clusterings, various evaluation measures have been developed over the years. There are two types of cluster evaluation measures: *external* and *internal* measures. The difference between the two is, that while the expected result – also known as *ground truth* – is known for the external measures, it is missing for the internal ones. Therefore, external evaluation measures make a qualitative comparison between the expected and the real result. Internal measures, however, focus on other describing characteristics, such as the compactness or separation of clusters in order to evaluate the quality of the result.

One common metric is the *Sum of Squared Errors* (SSE) that evaluates the compactness of clusters. In case of fuzzy clusterings this measure can be used by weighting the membership degrees. The SSE is based on the calculation of the overall distance between the members and the *centroid* of a cluster. The centroid usually describes the mean of all cluster members. Since this measure only considers the compactness of clusters, further validity measures have been developed, which evaluate the compactness as well as the separation. Common examples are the *Silhouette Coefficient* [47], *Davies-Bouldin Index* [12] or *Dunn Index* [14]. When considering fuzzy clusterings, there are for example validity measures which use only membership degrees [28, 35] or include the distances between data points and cluster prototypes [5, 7].

However, all these metrics cannot directly be compared to our method since they lack a temporal aspect, but they can be applied in our stability evaluation methods.

### Stability evaluation of clusterings

There are also several approaches addressing the stability measurement of a clustering algorithm. One example is the *Rand Index* [45], which is usually intended for the external evaluation of a clustering. Given the clustering *ζ*_*p*_ and the expected result *ζ*_*t*_, it examines on the one hand all object pairs that are located in the same cluster in *ζ*_*p*_ as well as *ζ*_*t*_, and on the other hand all pairs that belong to different clusters in both clusterings. The measure is defined by the number of corresponding object pairs in relation to the number of all possible object pairs.

The measurement of the stability of a clustering algorithm is for instance executed when searching for the optimal parameter setting. In 2002, Roth et. al [46] introduced the resampling approach for cluster validation. Roth et. al put forward the hypothesis, that if multiple partitionings of a clustering algorithm for the same parameter setting are similar to each other, the parameter setting is good. The higher the similarity, the better is the parameter choice.

The *unsupervised cluster stability value* *s*(*c*) that is used in Roth et. al’s approach [46] is calculated as the average pairwise distance between *m* partitionings:

2 $$ s(c) = \frac{\sum\limits_{i = 1}^{m -1} \sum\limits_{j = i + 1}^{m} d(U_{ci}, U_{cj})}{m\cdot (m - 1)/2}~, $$

where *U*_*ci*_ and *U*_*cj*_, 1 ≤ *i* < *j* ≤ *m*, are two partitionings produced for *c* clusters and *d*(*U*_*ci*_,*U*_*cj*_) is an arbitrary similarity index of partitionings. The Rand Index can be used for stability evaluation by including it in this formula. Such stability measures pursue a different objective and obviously do not take a temporal linkage into consideration [55]. Our stability measure is similar to the unsupervised cluster stability value but it includes the temporal dependencies of clusterings. An intuitive idea for achieving a temporal linkage would be to simply compare clustering pairs of successive points in time. This approach would strongly weight variation between two points in time and neglect long-term changes. An ongoing change would for instance be punished only slightly, since consecutive clusterings would be very similar, while short-term deviations would stand out, although the overall behavior might be stable. Also, the index would be strongly negatively affected by separations or merges of clusters of successive time points. Even when comparing clustering pairs of all different time points these problems would persist.

In addition, the referred methods exclusively evaluate the (over-time) stability of clusterings. As stated in [4, 32], however, stability alone is not sufficient for a proper evaluation of a clustering. CLOSE takes both into account, the over-time stability as well as the quality of a clustering, to give an overall rating for an over-time clustering.

### Anomaly detection in time series

When regarding works dealing with outlier detection in time series, various definitions of the term *outlier* can be found. Many approaches consider only single conspicuous data points such as additive outliers or change points [24, 37, 43] and focus on a single time series [1, 39]. However, in our context the detection of anomalous subsequences is considered, so that only algorithms, which either handle outlier subsequences or analyse the group behavior of multiple time series over time, are relevant.

For the latter, approaches such as Probabilistic Suffix Trees (PST) [49], Random Block Coordinate Descents (RBCD) [59] and various neural networks [26] have been developed and been shown to achieve convincing results. However, while these methods examine the deviation of one time series to all others in the data set, we focus on the behavior of a time series compared to its steady neighbors, since the consideration of the whole data set is only meaningful, if all TS have a similar course. This is for example the case in sensor data. In order to analyse the group behavior over time, we first have to identify continuous peers by clustering the TS data per time point. Then, the transitions of sequences between different clusters over time can be analysed. This type of transitions is also evaluated in cluster evolution methods. Landauer et al. [34] make use of such a method in order to calculate a prediction-based anomaly score for a single data point. Similar to our approach, the TS data is clustered per timestamp. The cluster transitions of a considered time series are then analysed by cluster evolution methods in order to approximate a model which predicts the next data point. Although groups of time series are identified, the detection of outliers is therefore based on the prediction of a single sequence. In contrast to Landauer et al. we refer to several time series.

Our approach is very different from clustering whole time series or their subsequences, since in that case the outlier detection relies on the single fact whether a sequence is assigned to a cluster or not. Such an approach does not take the cluster transitions of a sequence into account, which may be an expressive feature on its own. Hence, our approach might recognize anomalous subsequences which in a subsequence clustering would have been assigned to a cluster and therefore not been marked as outlier.

Apart from clustering subsequences, there are also other approaches for the detection of conspicuous subsequences or so called *discords* [25, 36]. Those often consider only a single time series at once. Therefore, only anomalous behavior with regard to the course of one sequence is recognized. Though, in the context of the whole data set, this behavior might for example be normal. Such methods are thus not applicable in our context.

## Methodology

The agglomeration of similar time series is a problem which arises in many applications. There are various approaches and a lot of research happened in this field. Since the definitions differ in related works, we first present our notations of relevant concepts for our work. Subsequently, we will describe the principles of our approaches CLOSE [51] and FCSETS [30].

### Notations

The following definitions are based on our previous works [30, 50, 51].

#### **Definition 1** (Data Set)

A data set *D* = {*T*_1_,...,*T*_*m*_} is a set of *m* time series of same length *n* and equivalent points in time. Equivalent means, that they are either identical or they can be mapped to a reference timestamp.

#### **Definition 2** (Time Series)

A time series $T = o_{t_{1}}, ... , o_{t_{n}}$ is an ordered set of *n* real valued data points of arbitrary dimension. The data points are chronologically ordered by their time of recording, with *t*_1_ and *t*_*n*_ indicating the first and last timestamp, respectively.

The vectors of all time series are denoted as the set $O = \{o_{t_{1},1},..., o_{t_{n},m}\}$, with the second index indicating the time series where this data point originates from. For the ease of reference, we write $O_{t_{i}}$ for all data points at a certain point in time.

#### **Definition 3** (Subsequence)

A subsequence $T_{t_{i}, t_{j}, l} = o_{t_{i}, l}, ... , o_{t_{j}, l}$ with *j* > *i* is an ordered set of successive real valued data points beginning at time *t*_*i*_ and ending at *t*_*j*_ from time series *T*_*l*_.

#### **Definition 4** (Cluster)

A cluster $C_{t_{i}, j} \subseteq O_{t_{i}}$ at time *t*_*i*_, with *j* ∈{1,...,*N*_*C*_} being a unique identifier (e.g. counter), is a set of similar data points, identified by a cluster algorithm, where *N*_*C*_ is the number of clusters. This means that all clusters have distinct labels regardless of time.

#### **Definition 5** (Cluster Member)

A data point $o_{t_{i}, l}$ at time *t*_*i*_, that is assigned to a cluster $C_{t_{i},j}$ is called a member of cluster $C_{t_{i},j}$.

#### **Definition 6** (Noise)

A data point $o_{t_{i}, l}$ at time *t*_*i*_ is considered as noise, if it is not assigned to any cluster. A data point that belongs to noise is also called an *outlier*. *Noise* describes the set of noise data points of all timestamps, i.e. $Noise = \bigcup _{k} Noise_{t_{k}}$.

#### **Definition 7** (Clustering)

A clustering is the overall result of a clustering algorithm for all timestamps. It is defined by the set $\zeta = \{C_{t_{1},1}, ..., C_{t_{n},N_{C}}\} \cup Noise$.

#### **Definition 8** (Time Clustering)

A time clustering is the result of a clustering algorithm at one timestamp. It is defined by the set $\zeta _{t_{k}} = \{C_{t_{k},a}, ..., C_{t_{k},b}\} \cup Noise_{t_{k}}$ of all clusters at time *t*_*k*_.

#### **Definition 9** (Fuzzy Cluster Membership)

The membership degree $u_{C_{t_{i},j}}(o_{t_{i},l}) \in [0,1]$ expresses the relative degree of belonging of the data object $o_{t_{i},l}$ of time series *T*_*l*_ to cluster $C_{t_{i},j}$ at time *t*_*i*_.

#### **Definition 10** (Fuzzy Time Clustering)

A fuzzy time clustering is the result of a fuzzy clustering algorithm at one timestamp. It is defined by the membership matrix $U_{t_{i}} = [u_{C_{t_{i},j}}(o_{t_{i},l})]$.

#### **Definition 11** (Fuzzy Clustering)

A fuzzy clustering of time series is the overall result of a fuzzy clustering algorithm for all timestamps. It is defined by the ordered set $U = U_{t_{1}}, ..., U_{t_{n}}$ of all membership matrices.

**Fig. 1:**
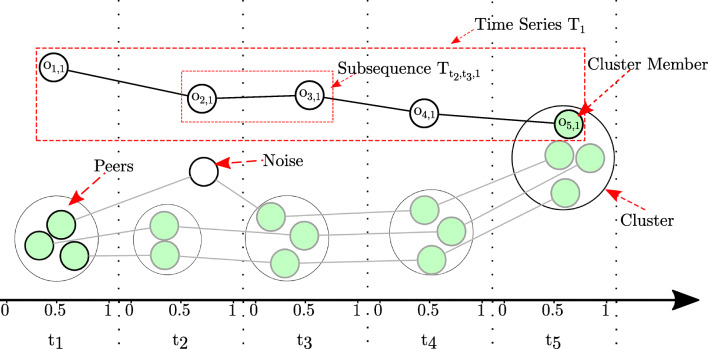
Illustration of the most important definitions. Lines between objects of a time series represent the development of the sequence [29]

An example for the above definitions can also be seen in Figs. 1 and 4. In Fig. 4, five time series of a data set *D* = *T*_*a*_, *T*_*b*_, *T*_*c*_, *T*_*d*_, *T*_*e*_ are clustered per timestamp for the time points *t*_*i*_,*t*_*j*_ and *t*_*k*_. The data points of a time series *T*_*l*_ are denoted by the identifier *l* for simplicity reasons. The shown clustering consists of six clusters. It can be described by the set $\zeta = \{C_{t_{i},l}, C_{t_{i}, u}, C_{t_{j},v}, C_{t_{j},f}, C_{t_{k},g}, C_{t_{k},h}\} \cup \{o_{t_{i},e}\}$. As $o_{t_{i},e}$ is not assigned to any cluster in *t*_*i*_, it is marked as noise for this timestamp. The data points $o_{t_{i},a}, o_{t_{i},b}$ of time series *T*_*a*_ and *T*_*b*_ in *t*_*i*_ are cluster members of the yellow cluster $C_{t_{i}, l}$. The subsequences $T_{t_{i},t_{j},a}$ and $T_{t_{i},t_{j},b}$ from time series *T*_*a*_ and *T*_*b*_ move both from the yellow ($C_{t_{i}, l}$) to the red ($C_{t_{j}, v}$) cluster. The green ($C_{t_{k},h}$) and pink ($C_{t_{k},g}$) cluster can be summarized by the time clustering $\zeta _{t_{k}}$ at time *t*_*k*_.

### Over-time stability evaluation

Since we want to measure the stability of an over-time clustering, whereby the partitioning may be produced by an arbitrary (evolutionary) clustering algorithm, we assume that different clusterings constitute different cluster connectedness based on the underlying TS members. Time series, which separate from their clusters’ members often, indicate a low over-time stability. For this reason, we first analyse the behaviour of every subsequence of a time series $T = o_{t_{1}}, ... o_{t_{k}}$, with *t*_*k*_ ≤ *t*_*n*_, starting at the first timestamp. In case of a hard clustering, subsequently, every cluster is rated by a stability function, based on the previous subsequence analysis of its members and the number of clusters that merged into the considered cluster. The final over-time stability score for the whole clustering can then be calculated with the rating of each cluster. When regarding fuzzy clusterings, the over-time clustering is directly rated based on the subsequence scores.

#### CLOSE

Given a TS data set *D* = {*T*_*l*_|1 ≤ *l* ≤ *m*} with *n* timestamps and an over-time clustering *ζ*, let $C_{t_{i}, a}$ and $C_{t_{j}, b}$ be two clusters of *ζ*, with *t*_*i*_,*t*_*j*_ ∈{*t*_1_,...*t*_*n*_}. The *temporal cluster intersection*, which is used for the stability evaluation of a subsequence, is defined as follows

3 $$ \cap_{t} \{C_{t_{i}, a}, C_{t_{j}, b}\} = \{T_{l} ~|~ o_{t_{i}, l} \in C_{t_{i}, a} \land o_{t_{j}, l} \in C_{t_{j}, b} \}~, $$

with *l* ∈{1,...,*m*}. The resulting set consists of time series, which contain data points that are grouped to the same cluster in *t*_*i*_ and *t*_*j*_. The transition of a subsequence from one cluster $C_{t_{i}, a}$ in *t*_*i*_ to another $C_{t_{j}, b}$ in *t*_*j*_ along with its group behaviour, which may be interpreted as *team spirit*, can now be expressed by the proportion of members of $C_{t_{i}, a}$ remaining together in *t*_*j*_

4 $$ p(C_{t_{i}, a}, C_{t_{j}, b}) = \begin{cases} 0 &  \text{if } C_{t_{i}, a} = \emptyset\\ \frac{|C_{t_{i}, a} \cap_{t} C_{t_{j}, b}|}{|C_{t_{i}, a}|} &  \text{else}\\ \end{cases}  $$

with *t*_*i*_ < *t*_*j*_. Regarding the example in Fig. 4 the proportion for $C_{t_{i}, l}$ and $C_{t_{j}, v}$ is defined by

$$ p(C_{t_i, l}, C_{t_j, v}) = \frac{|\{a, b\}|}{|\{a, b\}|} = \frac{2}{2} = 1.0~. $$

This proportion can be used to evaluate the over-time stability of a subsequence by rating its history with a *subsequence score*. In order to address the clusters a data point is assigned to, we first need to introduce an auxiliary function, which we call *cluster-identity function*:

5 $$ cid(o_{t_{i},j}) = \begin{cases} \emptyset &  \text{if the data point is not assigned to a cluster}\\ C_{t_{i},l} &  \text{else}\\ \end{cases} $$

For a data point $o_{t_{i},j}$ at time *t*_*i*_ the function returns the cluster it is assigned to. The subsequence score is then defined by

6 $$ subseq\_score(o_{t_{k},l}) = \frac{1}{k} \cdot \sum\limits_{i=1}^{k - 1} p(cid(o_{t_{i},l}), cid(o_{t_{k},l}))~,  $$

with *l* ∈{1,...,*m*} and *k* being the number of timestamps where the data point exists. That means, that all time points in which an object is an outlier, get the worst possible score of 0. The *subsequence score* takes into account how many cluster members of the object from the previous timestamps have migrated together over time.

In the example of Fig. 4, the score of time series *T*_*a*_ at time point *t*_*k*_ would be:

$$ subseq\_score(o_{t_k,a}) = \frac{1}{2} \cdot (\frac{2}{2} + \frac{2}{3}) = 0.83~. $$

This value reflects a quite high stability, which can be explained by the fact that *T*_*a*_ moves with most of its cluster members over the time period. The time series *d*, gets a significantly lower value of $subseq\_score(o_{t_{k},d}) = 0.5$, as it never moves with any of its cluster members. Note, that the impact of transitions of single TS becomes significantly lower when considering larger data sets.

The stability of a cluster can now be evaluated, focussing on two factors. The first one is the number of different clusters of previous timestamps, that merged into the regarded cluster. This can be expressed by

7 $$ m(C_{t_{k}, i}) = |\{C_{t_{l}, j} ~|~ t_{l} < t_{k} \land \exists a: o_{t_{l},a} \in C_{t_{l}, j} \land o_{t_{k},a} \in C_{t_{k}, i}\}|~, $$

Furthermore, a cluster’s stability score depends on the subsequence rating of all its cluster members. The second factor is therefore the sum of all *subsequence scores* of the data points within the considered cluster. Hence, the *over-time stability* of a cluster is defined as

8 $$ ot\_stability(C_{t_{k}, i}) = \frac{\frac{1}{|C_{t_{k}, i}|} \cdot {\sum}_{o_{t_{k},l} \in C_{t_{k},i}} subseq\_score(o_{t_{k}, l})}{\frac{1}{k - 1} \cdot m(C_{t_{k},i})} $$

for *k* > 1. For a cluster at time point *t*_*k*_, the entire preceding time frame [*t*_1_,*t*_*k*− 1_] is considered. We define clusters at the first timestamp to be stable and set $ot\_stability(C_{t_{1}, i}) = 1.0$. In order to make clusters comparable, the sum of *subseq_score* is averaged by the number of data points in the viewed cluster, while the number of merged clusters is averaged by the number of timestamps before the regarded cluster. There are clustering algorithms which do not assign a cluster to every data point. Those data points are usually denoted as outliers. It is important to mention, that the number of merged clusters does not take these outliers into account.

Regarding the example of Fig. 4, the stability of the cluster $C_{t_{k},g}$ is given by:

$$ ot\_stability(C_{t_k, g}) = \frac{\frac{1}{3} \cdot (0.83 + 0.58 + 0.25)}{\frac{1}{2} \cdot 4} = 0.28~. $$

This low score can be explained by the fact that the cluster under consideration contains only three data points. One of those (*T*_*e*_) has a completely independent course of its clusters’ members, and the remaining two are not perfectly stable either.

Finally, the over-time stability of a clustering *ζ* can be calculated by

9 $$ \begin{array}{@{}rcl@{}} CLOSE(\zeta) &=& \frac{1}{N_{C}} \cdot \Big(1 - \Big(\frac{n}{N_{C}}\Big)^{2}\Big) \cdot \Big( \sum\limits_{C \in \zeta} ot\_stability(C)\\ &&\cdot (1 - quality(C))\Big)~, \end{array} $$

with *N*_*C*_ being the number of clusters of the over-time clustering *ζ*, *n* being the number of timestamps and *quality* being an arbitrary cluster evaluation measure. When working with normalised data ∈ [0,1]^*d*^, we suggest the mean squared error (MSE), but any other rating function can also be used. Please make sure of using a function, whose results lie in the interval of [0,1] in order to get appropriate results. When using a function for evaluating the quality instead of the deficiency of a clustering – that means, higher values indicate a higher quality – the term (1 − *quality*(*C*)) may e.g. be replaced by (1 − *quality*(*C*)^− 1^) or *quality*(*C*) depending on the quality measure.

As long as the output of the quality function is between 0 and 1 and there exists at least one cluster per timestamp, CLOSE as well returns a score between 0 and 1, with 1 indicating a good over-time clustering.

The first pre-factor results from averaging by the number of clusters. The second factor $1 - (\frac {n}{N_{C}})^{2}$ is intended to counteract one large cluster to get a high score. Since such a clustering automatically exhibits a very high over-time stability, the CLOSE score rises. Note, that the clusters of the first point in time are also included in the evaluation measure. Since they are assumed to have a stability of 1.0, the score is in general slightly increased and for the first timestamp only influenced by the quality of the clusters.

##### *Remark 1* (Time Point Comparison)

In contrast to the evaluation function integrated in evolutionary clustering [8, 27, 58], where only consecutive points in time are compared, CLOSE compares clusterings of all preceding time points with the last timestamp of the considered subsequence. This has multiple effects. First, the stability score is robust against outliers. Second, short-term transitions between clusters are weighted more lightly. Simultaneously, long-term changes that develop slowly over time are punished more severely, which forms the third effect. Note: The formula cannot be transformed to simply iterate over all cluster pairs. Since the *over-time stability* is weighted with the *quality* of the cluster, the results would differ.

##### *Remark 2* (Handling Outliers)

Our calculations are suitable for both cleaned data and data with noise. Currently, outliers have only a minor impact on the score. That is, because they are solely considered in the subsequence score and not in the cluster stability. However, apart from decreasing the subsequence score, they have an additional indirect influence on the clustering score. Since the pre-factor in Formula (9) favours a large number of clusters, it may be more advantageous for the clustering algorithm to assign data points to smaller clusters than to interpret them as noise and recognize only a few large clusters.

This weak treatment of outliers is reasoned considering the idea, that the over-time clustering might be used for outlier detection. In this case, the algorithm should not be pushed into assigning every data object to a cluster. Nevertheless, different strategies for treating outliers might be investigated in future work.

One way to penalize noise more strongly would be, to insert an *exploitation term* which represents the number of data points that are assigned to a cluster *N*_*co*_ in relation to the number of all existing data points *N*_*o*_. In order to achieve high CLOSE scores, this term should be maximized then. The formula including the exploitation term is given by

10 $$ \begin{array}{@{}rcl@{}} CLOSE(\zeta) &=& \frac{1}{N_{C}} \cdot \Big(1 - \Big(\frac{n}{N_{C}}\Big)^{2}\Big) \cdot \Big(\sum\limits_{C \in \zeta} ot\_stability(C)\\ &&\cdot (1 - quality(C))\Big) \cdot \frac{N_{co}}{N_{o}}~, \end{array} $$

##### *Remark 3* (Merge & Split of Clusters)

Considering the *subsequence score* (Formula (6)), a merge of clusters do not have a negative impact on the score. On the contrary: if two clusters fuse entirely, the score is actually increased, as all objects move together with all their cluster members and therefore show a good team spirit. This is intended, since the focus lies primarily on the cohesion of time series. A good team spirit is rewarded in every case.

When considering cluster splits, though, the *subsequence score* is lowered. Since a split indicates that time series which have been members of the same cluster at some point in time separate from each other, this behaviour is also wanted. Note, that in the case, where smaller clusters have previously merged together and then separated again in the same way as before, the influence on the score is not high and vanishes over time.

However, in some applications the punishment of cluster merges might be desired. As we will show in Section 4 regarding our proposed outlier detection algorithm, the Jaccard Index can be used in the proportion calculation, in order to penalize merges and splits in the same way.

##### *Remark 4* (Additional Remarks)

As Ben et al. stated, the sample size has a high impact on the stability evaluation of a clustering [4]. This is not only the case, when considering constant data points. When examining the over-time stability of a clustering, a small sample size also leads to a high sensitivity to transitions between clusters. The greater the considered data set, the easier a statement about the (over-time) stability can be made. In order to extend the method for a broader field of quality measures, the formula of CLOSE can be modified, so that quality measures for clusterings instead of clusters can be used. Therefore, the average cluster stability *avg_stab* per time clustering $\zeta _{t_{i}}$ must be considered. The score is then normalised using the number of timestamps *n*:

11 $$ CLOSE(\zeta) = \frac{1}{n} \cdot \Big(1 - \Big(\frac{n}{N_{C}}\Big)^{2}\Big) \cdot \Big(\sum\limits_{\zeta_{t_{i}} \subset \zeta} avg\_stab(\zeta_{t_{i}}) \cdot (1 - quality(\zeta_{t_{i}}))\Big)~. $$

**Fig. 2:**
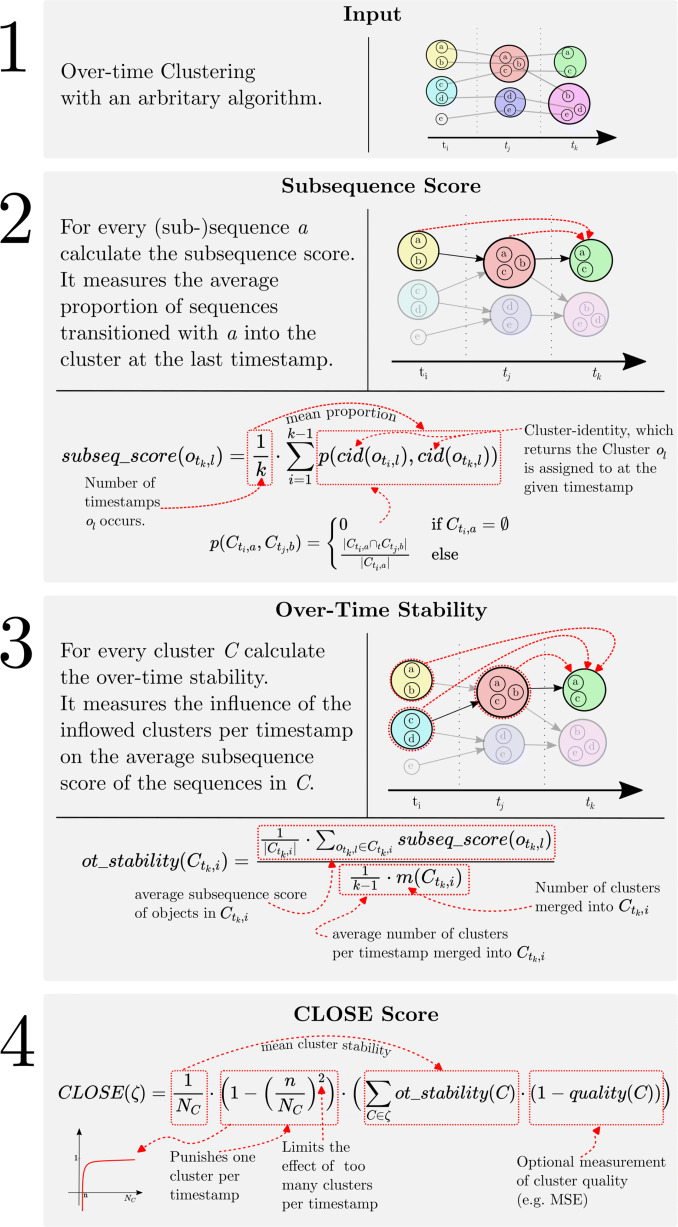
Detailed step-by-step explanation of CLOSE

Figure 2 summarises the calculation process and explains the most important formulas.

#### FCSETS

Given a TS data set *D* = {*T*_*i*_|1 ≤ *i* ≤ *m*} with *n* timestamps and a fuzzy over-time clustering *U*. Let $U_{t_{i}} \subset U$ be a fuzzy partitioning of the data objects $O_{t_{i}}$ of all times series at time *t*_*i*_ in $k_{t_{i}}$ clusters. The relative assignment agreement of two data objects $o_{t_{i},l}$ and $o_{t_{i},s}$ from time series *T*_*l*_ and *T*_*s*_ to all clusters in the partitioning $U_{t_{i}}$ at time *t*_*i*_ can be calculated using the equivalence relation from Hüllermeier-Rifqi Index (HRI) [20]:

12 $$ E_{U_{t_{i}}}(o_{t_{i},l}, o_{t_{i},s}) = 1 - \frac{1}{2} \sum\limits_{j = 1}^{k_{t_{i}}}|u_{C_{t_{i},j}}(o_{t_{i},l}) - u_{C_{t_{i},j}}(o_{t_{i},s})|~,  $$

with $u_{C_{t_{i},j}}(o_{t_{i},l})$ being the membership degree of the data point $o_{t_{i},l}$ regarding the cluster $C_{t_{i},j}$ (see Definition 9). In order to measure the relation of two time series *T*_*l*_ and *T*_*s*_, we calculate the difference between their relative assignment agreements by subtracting the relative assignment agreement values:

13 $$ D_{t_{i},t_{r}}(T_{l}, T_{s}) = |E_{U_{t_{i}}}(o_{t_{i},l}, o_{t_{i},s}) - E_{U_{t_{r}}}(o_{t_{r},l}, o_{t_{r},s})|~. $$

Leaning on the Hüllermeier-Rifqi Index [20] – which deals with a slightly different task by calculating the normalised degree of concordance between two partitions – we define the over-time stability of a time series *T*_*l*_ as the average weighted difference between the relative assignment agreements to all other time series:

14 $$ stability(T_{l}) = 1 - \frac{2}{n(n-1)} \sum\limits_{i = 1}^{n-1} \sum\limits_{r = i + 1}^{n} \frac{\sum\limits_{s = 1}^{m} E_{U_{t_{i}}}(o_{t_{i},l}, o_{t_{i},s})^{m} D_{t_{i},t_{r}}(T_{l}, T_{s})^{2}}{\sum\limits_{s = 1}^{m} E_{U_{t_{i}}}(o_{t_{i},l}, o_{t_{i},s})^{m}}~. $$

The difference between the assignment agreements $D_{t_{i},t_{r}}(T_{l}, T_{s})$ is weighted by the assignment agreement between pairs of TS at a previous time point in order to damp large differences for stable time series caused by supervention of new peers. On the other hand, time series that leave their cluster peers when changing their cluster membership are penalized.

The over-time stability of a fuzzy clustering *U* can now be expressed by the average over-time stability of all time series in the data set:

15 $$ FCSETS(U) = \frac{1}{m} \sum\limits_{l = 1}^{m} stability(T_{l}). $$

**Fig. 3:**
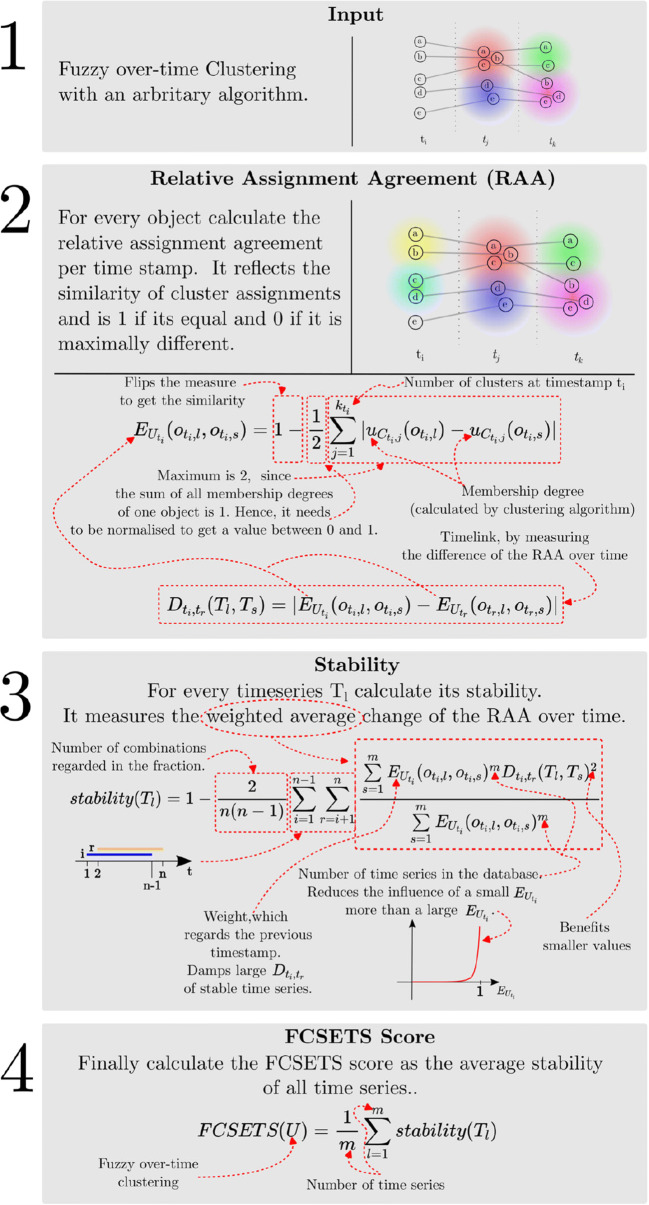
Detailed step-by-step explanation of FCSETS

A more efficient approach as a substitute for the HRI proposed by Runkler [48] is the *Subset Similarity Index (SSI)*. The efficiency gain is reasoned by the similarity calculation, which in SSI considers cluster pairs while HRI concentrates on the assignment agreement of data point pairs. In our context, where the clustering should be used for further analysis such as outlier detection, we aim to describe the over-time stability of clustering by the *team spirit* of the considered time series. Therefore, we believe, that the degree of the assignment agreement between TS pairs to clusters at different timestamps provide a greater information gain than the similarity between cluster pairs. For this reason, the SSI is not suitable for our over-stability evaluation. Figure 3 summarises the calculation process and explains the most important formulas.

## Applications

Our evaluation measures can not only be used for the over-time stability evaluation of clusterings, but also for further analyses such as parameter selection or outlier detection [50, 52, 53]. Therefore, for example the part of CLOSE, where subsequences are evaluated, can be used.

In [50], we present an approach called DOOTS (**D** etecting **O** utliers regarding their **O**ver-**T**ime **S**tability) for finding conspicuous subsequences of all lengths with an underlying over-time clustering regarding the following definition:

### **Definition 12** (Anomalous Subsequence)

A subsequence $T_{t_{i}, t_{j}, l}$ is called anomalous, if it is significantly more unstable than its cluster members at time *t*_*j*_.

For this, the subsequence score from Formula (6) has to be reformulated in order to handle subsequences with arbitrary starting points. The *subsequence score* of a subsequence $T_{t_{i},t_{j},l}$ of time series *T*_*l*_ starting at *t*_*i*_ and ending at *t*_*j*_ is defined as

16 $$ subsequence\_score(T_{t_{i}, t_{j},l}) = \frac{1}{k} \cdot \sum\limits_{v=i}^{j - 1} p(cid(o_{t_{v},l}), cid(o_{t_{j},l})) $$

with *l* ∈{1,...,*m*}, *k* ∈ [1,*j* − *i*] being the number of timestamps between *t*_*i*_ and *t*_*j*_ where the time series exists [50].

One noteworthy aspect is that the score is always 0, if the last data point of the considered subsequence is marked as noise. In most cases, this does not lead to any handicaps regarding the analysis, since all partial sequences of these subsequences are treated normally, though. Nevertheless, a more detailed discussion of such situations will be provided in the further course of this work.

**Fig. 4:**
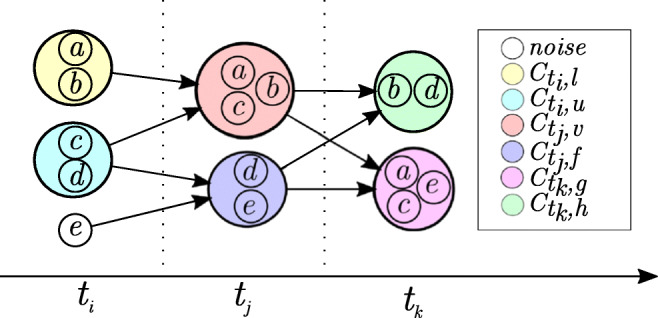
Example for cluster transitions of time series *T*_*a*_,..,*T*_*e*_ over time [52]

As already mentioned, the used *proportion* from Formula (4) is asymmetric and punishes splits while ignoring merges. In order to counteract this circumstance, the jaccard index can be used, as proposed in [52]. Therefore, the *temporal cluster union* of two clusters $C_{t_{i}, a}, C_{t_{j}, b}$ has to be introduced first:

17 $$ \cup_{t} \{C_{t_{i}, a}, C_{t_{j}, b}\} = \{T_{l} ~|~ o_{t_{i}, l} \in C_{t_{i}, a} \lor o_{t_{j}, l} \in C_{t_{j}, b} \} $$

with *l* ∈{1,...,*m*}. The proportion $\hat {p}$ can then be expressed by the jaccard index of two clusters:

18 $$ \hat{p}(C_{t_{i}, a}, C_{t_{j}, b}) = \begin{cases} 0 &  \text{if } C_{t_{i}, a} = \emptyset \land C_{t_{j}, b} = \emptyset\\ \frac{|C_{t_{i}, a} \cap_{t} C_{t_{j}, b}|}{|C_{t_{i}, a} \cup_{t} C_{t_{j}, b}|} &  \text{else}\\ \end{cases} $$

with *t*_*i*_ < *t*_*j*_. In contrast to the proportion from Formula (4) regarding the example in Fig. 4 the jaccard proportion is

$$ \hat{p}(C_{t_i, l}, C_{t_j, v}) = \frac{|\{a, b\}|}{|\{a, b, c\}|} = \frac{2}{3} = 0.67 $$

since the merge of (parts of) the yellow ($C_{t_{i}, l}$) and turquoise ($C_{t_{i},u}$) cluster gets punished.

Another characteristic of the subsequence score from CLOSE (Formula (6)) is the equal impact of all considered timestamps regarding the over-time stability of a subsequence. When considering longer sequences, however, this may lead to a tendency towards a worse rating, since slow changes in cluster memberships might influence the score considerably. Assuming that the nearer past is more significant than the more distant past, a weighting function can be integrated in the subsequence score.

Using the Gauss’ Formula, the weighting of the proportion at time *t*_*i*_ regarding the time interval [*t*_1_,*t*_*k*_] can be calculated by

19 $$ \frac{i}{{\sum}_{a=1}^{k} a} = \frac{i}{\frac{k (k + 1)}{2}} = \frac{2\cdot i}{k (k+1)}~. $$

Adjusting this weighting function to a time interval with arbitrary starting point *t*_*s*_ ≥ *t*_1_, the subsequence score is then defined by

20 $$ weighted\_subseq\_score(T_{t_{i}, t_{j},l}) = \sum\limits_{v=i}^{j - 1} \frac{2 \cdot (v - i + 1)}{k (k + 1)} p(cid(o_{t_{v},l}), cid(o_{t_{j},l}))~. $$

with *k* ∈ [1,*j* − *i*] again being the number of timestamps between *t*_*i*_ and *t*_*j*_ where the considered time series exists [52]. There is no need to normalize the score to an interval of [0,1] by averaging it, as the sum of all weightings of a subsequence’s timestamps is always 1 due the division by the Gauss’s Formula.

In contrast to the subsequence score, regarding the example in Fig. 4 the weighted subsequence score is given by

$$ subseq\_score(o_{t_k,a}) = \frac{1}{3} \cdot \frac{1}{2} + \frac{2}{3} \cdot \frac{2}{3} = 0.61 $$

which is a bit higher, since the immediately preceding (higher) score gets a greater weighting than the more distant one.

In summary, four options can be used: (i) the ordinary subsequence score (DOOTS), (ii) the weighted subsequence score (wDOOTS), (iii) the ordinary subsequence score using the jaccard proportion (jDOOTS) and (iv) the weighted subsequence score using the jaccard proportion (jwDOOTS).

With this score, a subsequence can now be compared with its cluster members, in order to determine, if its over-time stability stands out. In this respect we consider the following assumptions:

### *Assumption 1*

If the score of a subsequence is significantly lower than those of its cluster members, its over-time behavior is conspicuous.

### *Assumption 2*

If the score of a subsequence is low, but so are those of its cluster members, its over-time behavior is not conspicuous, since this low over-time stability shows a pattern of regularity.

In order to find outlier sequences of all lengths, every possible subsequence receives an outlier score indicating the probability of being anomalous. The outlier score describes the deviation of a subsequence’s stability from the best subsequence score of its cluster. Figuratively, one can imagine that the time series with the highest subsequence score represents a kind of leader and that a large deviation from this leader is to be considered conspicuous. The best subsequence score of a cluster $C_{t_{j}, a}$ regarding subsequences starting at time *t*_*i*_ is expressed by the following formula:

21 $$ best\_score(t_{i}, C_{t_{j}, a}) = max(\{subsequence\_score(T_{t_{i}, t_{j}, l}) ~|~ cid(o_{t_{j}, l}) = C_{t_{j}, a}\}) $$

The outlier score can then be calculated by

22 $$ \begin{array}{@{}rcl@{}} outlier\_score(T_{t_{i}, t_{j}, l}) &=& best\_score(t_{i}, cid(o_{t_{j}, l}))\\ &&- subsequence\_score(T_{t_{i}, t_{j}, l})~. \end{array} $$

With respect to Assumptions 1 and 2, the outlier score depends on the best score of a cluster’s members. Therefore, an outlier score of 100*%* can only be achieved in clusters consisting exclusively of completely stable subsequences. On the other hand, a cluster with small stabilities only, can lead to a situation where no subsequence score is considered conspicuous, no matter how low it is. As mentioned in Assumption 2, this behavior is desired.

Using the outlier score and a threshold parameter *τ*, a more precise definition of an outlier can now be given.

### **Definition 13** (Outlier)

Given a threshold *τ* ∈ [0,1], a subsequence $T_{t_{i}, t_{j}, l}$ is called an outlier, if its probability of being an outlier is greater than or equal *τ*. That means, if

$$ outlier\_score(T_{t_{i}, t_{j}, l}) \geq \tau~. $$

Even though the parameter *τ* is constant, it can be considered as a dynamic threshold, since the greatest possible deviation from the best subsequence score – and simultaneously the greatest outlier score – is dependent on the best score of the considered cluster. Leaning on Assumption 2, clusters which show a low stability have a lower probability of containing an outlier than stable ones, because all their cluster members exhibit irregularities, which represents a pattern of instability. Thus, in this case, a small subsequence score is not conspicuous.

Subsequences that consist entirely of noise data points are automatically identified as outliers and are called *intuitive outliers*. This special treatment is needed, since subsequences whose last data point is labeled as noise do not have any cluster members which the best score can be calculated from. Therefore, no outlier score can be determined for them. Hence, in our outlier detection we consider three types of outliers: *anomalous subsequences* regarding Definition 13, *intuitive outliers* and data points marked as *noise* by a clustering algorithm.

Imagine examining a subsequence $T_{t_{i}, t_{j}, l}$ whose last data point at time *t*_*j*_ is marked as noise. In addition suppose its subsequence $T_{t_{i}, t_{j-1}, l}$ getting a high outlier score and therefore being detected as an outlier. Intuitively, one would expect the subsequence under consideration $T_{t_{i}, t_{j}, l}$ being identified as an outlier as well. In our approach, this would only be the case, if the sequence was recognized as an intuitive outlier i.e. the previous data point was categorized as noise, too. Anyway, the subsequence $T_{t_{i}, t_{k}, l}$ with *k* > *j*, which for the first time is assigned to a cluster again at its last time point *t*_*k*_, would be detected as an outlier. Thus, in the end $T_{t_{i}, t_{j}, l}$ would be covered.

Still, in the marginal case where a data point is labeled as noise at the last time of the entire time series, a subsequence with end time *t*_*m*_ would never be detected as an outlier, if it is not marked as noise in *t*_*m*− 1_. This drawback should be investigated in future works.

### *Remark 5* (Modifications)

As DOOTS is leaned on the presented evaluation measure, the modification of the proportion calculation using the Jaccard index as well as the weighting function for the *subsequence_score* may naturally also be applied to CLOSE, if desired.

## Evaluation

In this section, we present several experiments. First, we describe the different data sets, which we use in order to illustrate our results. Then we present clusterings calculated with K-Means [38] and DBSCAN [16]. In order to create those clusterings, we use common methods to identify good parameters per timestamp. Afterwards, we compare the results with clusterings whose parameters were identified with the help of CLOSE. These results are then compared to those of the evolutionary clustering presented in [8]. We also evaluate clusterings retrieved by Fuzzy C-Means [6] and focus on the achieved FCSETS scores. Finally, the comparison of clusterings is followed by applications to the outlier detection algorithm. We finish the section with qualitative analyses of the results.

### Data sets

In the following, we present the three data sets our analyses are based on.

#### COVID-19 data set

The COVID-19 pandemic is currently affecting the whole world. In this context, the hashtag #FlattenTheCurve is intended to encourage people all over the world to behave in a way that prevents the distribution of infections over time and thus counteracts overloading of the health care systems. Although the hashtag is used in an inflationary way, few people realise that the curve is actually a time series. Because of the current relevance of the data set, it is an excellent candidate for applying our methods. We obtained the data from the official GitHub repository of Johns Hopkins University^3^. Specifically, we used the daily reports on worldwide COVID-19 infections for our analyses. Depending on the country, the data set contains data on the individual regions (such as federal states) of the country concerned. We have aggregated these data so that for each available country, only one entry per point in time has been created. Over time, other features such as incidence were added. In order to provide the incidence for all points in time, we calculated the incidence using population data for the countries. For this purpose, we have obtained the population data for the respective countries from theglobaleconomy.com^4^. We then calculated the seven-day incidence for the countries. The incidence reflects the number of infections in the last seven days per 100,000 inhabitants. Due to the low infection figures at the beginning of the pandemic, the incidence value is particularly low at some times. For this reason, we give the number of infections per 10,000,000 inhabitants. In addition, we do not consider directly consecutive days, because the fluctuation in these is relatively small. Instead, we look at every seventh day, reflecting the development within a week.

#### ElectricityLoadDiagrams20112014 data set

The ElectricityLoadDiagrams20112014 dataset is from the UCI Machine Learning Repository [13]. It contains data on the electricity consumption of 370 customers at quarter-hourly intervals. We have summarised the electricity consumption into monthly intervals and selected the first 30 customers for a better overview. Summarising on a monthly basis also has the advantage that the resulting dataset has no missing values. For better comparability, we have also applied a min-max scaling to the data.

#### TheGlobalEconomy.com data set

We extracted this data set from theglobaleconomy.com[2]. The website offers over 400 indicators on 200 countries for over 80 years. The indicators include data such as GDP, inflation, population data, employment rates and many more. All available data have been obtained from reliable official sources. From the large number of available indicators, we selected two for illustration purposes, namely the unemployment rate and the education spending. The two features are on the one hand the educational expenditure and on the other hand the unemployment rate. In addition, we have only considered twenty countries for the purpose of the overview.

#### Generated data set

In order to show specific characteristics of CLOSE and our outlier detection algorithm, we generated two artificial data sets. The first contains 40 time series with 6 time points and two-dimensional feature vectors in [0,1]^2^. For every timestamp, four cluster centroids have been set, which 10 time series were assigned to with a maximal distance of 0.1 each. The cluster members remain the same for the whole time period, but the clusters merge and split over time. More precisely, at any time point only three clusters are visible, since at the moment where one cluster splits (*t*_4_), two others merge into one.

For the evaluation of our outlier detection algorithm, three transition-based outliers have been inserted in the data set. For each timestamp, the outlier sequences have been randomly assigned to a cluster centroid with a maximal distance of 0.1.

### Density-based clustering

Since to the best of our knowledge there are no other evaluation measures for the over-time stability of clusterings-per-timestamp, a quantitative evaluation against other measures is not possible. The comparison to other common stability measures is not meaningful either, as the targeted stability definition differs. Nevertheless, the evaluation of clusterings retrieved with parameter settings determined by CLOSE against those of evolutionary clustering algorithms, may surrogate such an analysis as the objective function which is optimized in evolutionary clustering includes a similar definition of over-time stability. Apart from the comparison with evolutionary clusterings, our evaluation section deals with different experiments on real world and artificially generated data sets in order to discuss different characteristics of CLOSE and its applications.

**Fig. 5:**
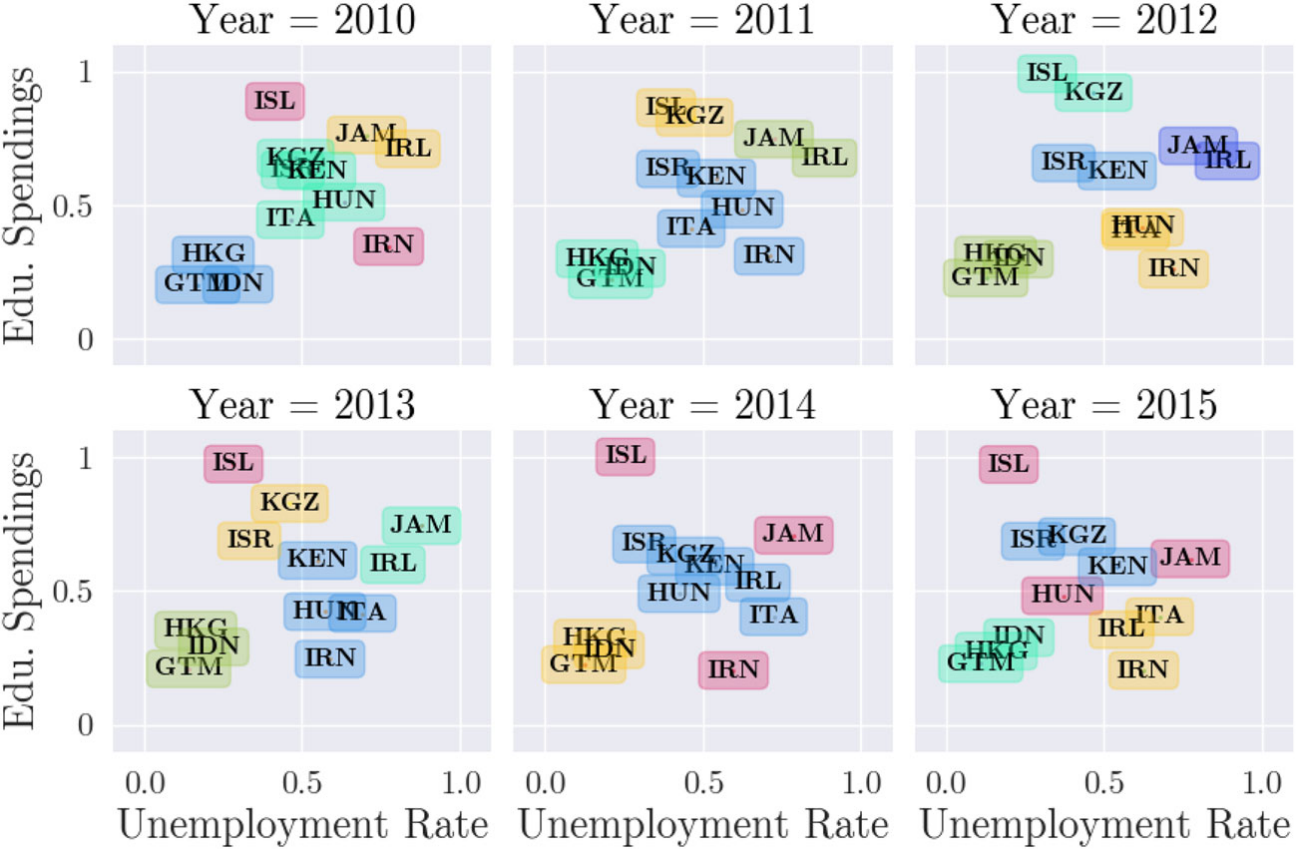
Best resulting clustering with DBSCAN (*𝜖* = 0.2, *minPts* = 2) achieving a CLOSE score of 0.514 on the GlobalEconomy data set

**Fig. 6:**
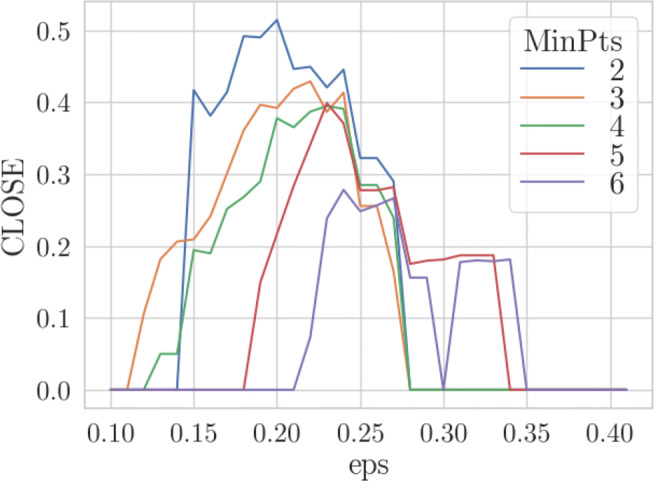
Resulting CLOSE score for different *minPts* depending on *𝜖*

In the first experiment we investigate the behavior of the CLOSE score depending on the parameter setting of DBSCAN regarding the GlobalEconomy data set. In Fig. 6 this behavior is illustrated. For each *minPts* a colored line is drawn, which shows the CLOSE score depending on *𝜖*. We tested all *minPts* ∈ [2,6] and *𝜖* ∈ [0.1,0.4] with a step size of 0.01. The best result was achieved with *minPts* = 2 and *𝜖* = 0.2 and is shown in Fig. 5.

As can be seen, the resulting clustering is quiet stable although the data set is rather dispersed and some of its data objects have irregular movements. For example, Jamaica (JAM) and Ireland (IRL) are completely stable over time as they are always together in one cluster. Such a stability can only be achieved with *minPts* = 2 since bigger clusters would lead to more cluster transitions. This characteristic can also be read off the diagram in Fig. 6, where the curve of *minPts* = 2 reaches higher CLOSE scores than the others in most cases. Obviously, regarding this data set, it is difficult to determine *one* optimal *𝜖* since the groups of objects move towards each other. The choice of one fix parameter setting leads for example to the creation of a single cluster in the last considered timestamp. Although it is not desired to have only one cluster, since it does not lead to a high information gain, it is an intuitive result in this case, though. When choosing a smaller *𝜖* in order to counteract this circumstance, the over-time stability would be significantly decreased.

When considering the line of *minPts* = 6 in Fig. 6, the results might seem unintuitive since the CLOSE score is 0 for most of the time and it gets higher with *𝜖* > 0.3 although it already reached a score of 0 before. The first characteristic can be explained by the high *minPts* value since *𝜖* has to be chosen relatively high in order to reach enough data points to put together in one cluster. The second characteristic is caused by the pre-factor of CLOSE which sets the score to 0, if there are not at least *k* clusters, where *k* is the number of timestamps. For *𝜖* = 0.3 only one cluster per timestamp is found which causes a high amount of outliers. By increasing *𝜖* new clusters are created, whose members have been marked as noise for lower *𝜖*. This applies in particular to the years 2012 and 2013.

**Fig. 7:**
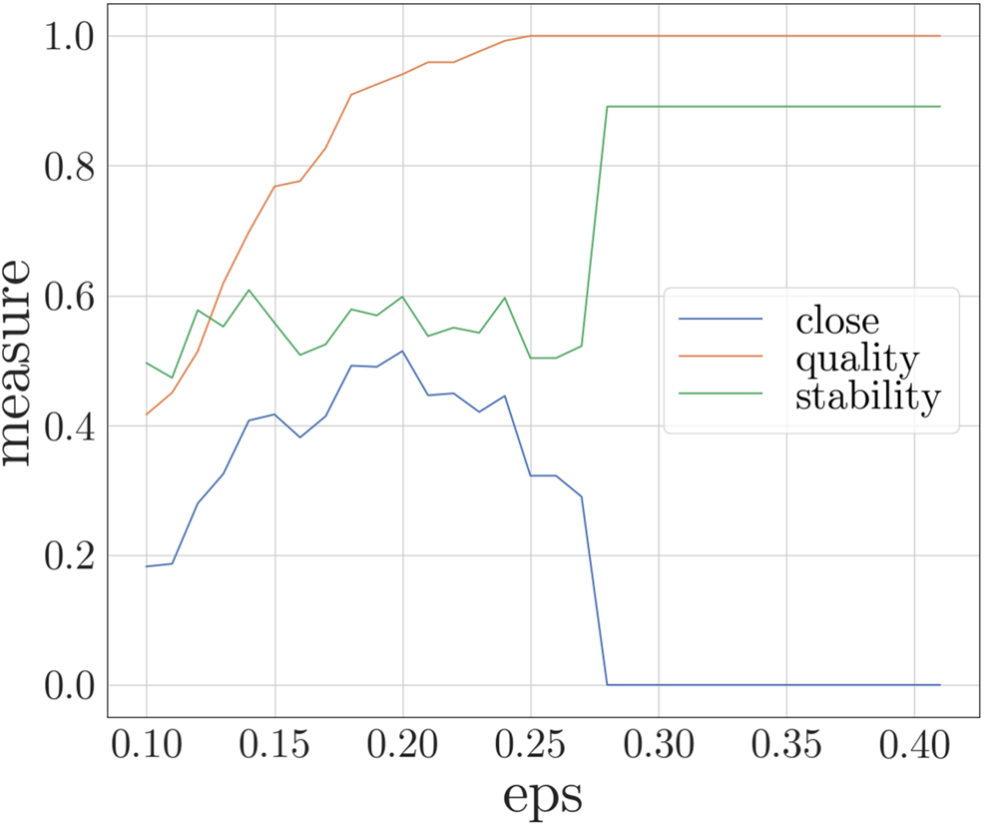
Resulting CLOSE score, stability and quality for *minPts* = 2 depending on *𝜖*

In Fig. 7 the behavior of the *ot_stability*, *quality* and the CLOSE score (see Formula (9)) depending on *𝜖* can be compared. *minPts* was set to 2, as it proved to be the best choice on the GlobalEconomy data set. The quality was measured by the amount of objects that are assigned to a cluster in relation to all objects at the considered time point. The usage of such a simple measure can be justified by the fact that the density of the resulting clusters is already indirectly evaluated by the clustering algorithm DBSCAN. Also, evaluation measures addressing the separation and compactness of clusters are not suitable for density-based clustering algorithms. Therefore, the aim is to minimize the amount of outliers as they are not caught in the formula of CLOSE. The diagram shows that, as long as the quality is lower than the stability (*𝜖* ≤ 0.13), it has a high impact on the CLOSE score. Afterwards, the curve of CLOSE is very similar to the stability. For *𝜖* > 0.26 the CLOSE score gets worse, although the quality as well as the stability increases. The CLOSE score decreases rapidly to 0, which is caused by the fact, that the number of clusters falls below the number of timestamps. In other cases the score would highly depend on the number of clusters as long as they exceed the number of timestamps, if the quality and stability remain almost the same.

### K-Means

In this paragraph we compare the achievable CLOSE score of K-Means with those of the evolutionary K-Means of [8]. For this, we first used the one-dimensional COVID-19 data set. The evolutionary clustering approach from [8] softens the definition of partitioning clustering: At a point in time, the space is classically partitioned into *k* regions, but the assignment of individual elements to a cluster is also based on the partitionings of the previous points in time. The assignment function is therefore based on two components, the so-called history costs and the distance to the cluster centre. The user must specify a weighting for these two components in advance. In addition, this approach requires an unknown function *f* that maps clusters from two points in time to each other. Although this function seems intuitive at first glance, it constitutes a separate field of research. Despite the problems mentioned above, evolutionary clustering has a decisive advantage that becomes more relevant when calculating stability. The assignment function of evolutionary clustering from [8] can assign objects to a cluster even if they lie in a different cluster from the point of view of a classical partitioning method. This can positively influence the stability of time series, clusters and thus also clusterings.

An adaptation of the classical K-Means to previous points in time can be realised with the help of varying *k* s. A search for the most stable clustering with varying *k* s is also possible with CLOSE, but we consider this scenario impractical because the number of configurations to be tested would increase considerably: For 10 time points and a *k* ∈ [2,5], this would already be 4^10^ = 1048576 combinations. A corresponding evaluation of the stability for time-dependent *k* would therefore be difficult to realise. For this reason, we search for one *k* that fits best for all time points. The clustering that achieves the highest CLOSE score is then compared with evolutionary clustering. In the following evaluations, the asymmetric proportion and the mean squared error as quality measure were used.

#### K-means and evolutionary K-means applied to the COVID-19 data set

The results of the two clustering algorithms applied to the COVID-19 data set are very different. First, the best *k* was identified for both approaches using CLOSE. Here, all *k* s in the interval of [2,10] were examined. For both algorithms, *k* = 4 was identified as the *k* that leads to the most stable clustering.

**Fig. 8:**
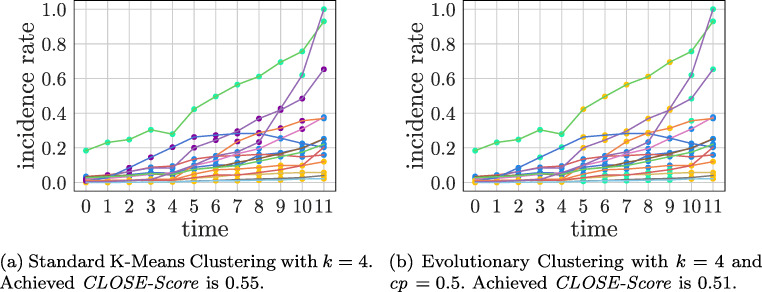
K-Means and evolutionary K-Means applied to the COVID-19 Data Set

For the evolutionary approach, the *change parameter* was set to 0.5. The results can be viewed in Fig. 8. The differences are particularly striking at times five to seven. These can be explained by the previously extended assignment function of the evolutionary approach. In this specific case, however, the evolutionary approach does not lead to a higher CLOSE score than the classical approach. Specifically, the standard approach produces a clustering that is 0.04 more stable than the evolutionary approach. This may not be a big difference, but it shows that the adjustments from [8] made for the evolutionary approach do not necessarily lead to better CLOSE score.

#### K-means and evolutionary K-means applied to the generated data set

**Fig. 9:**
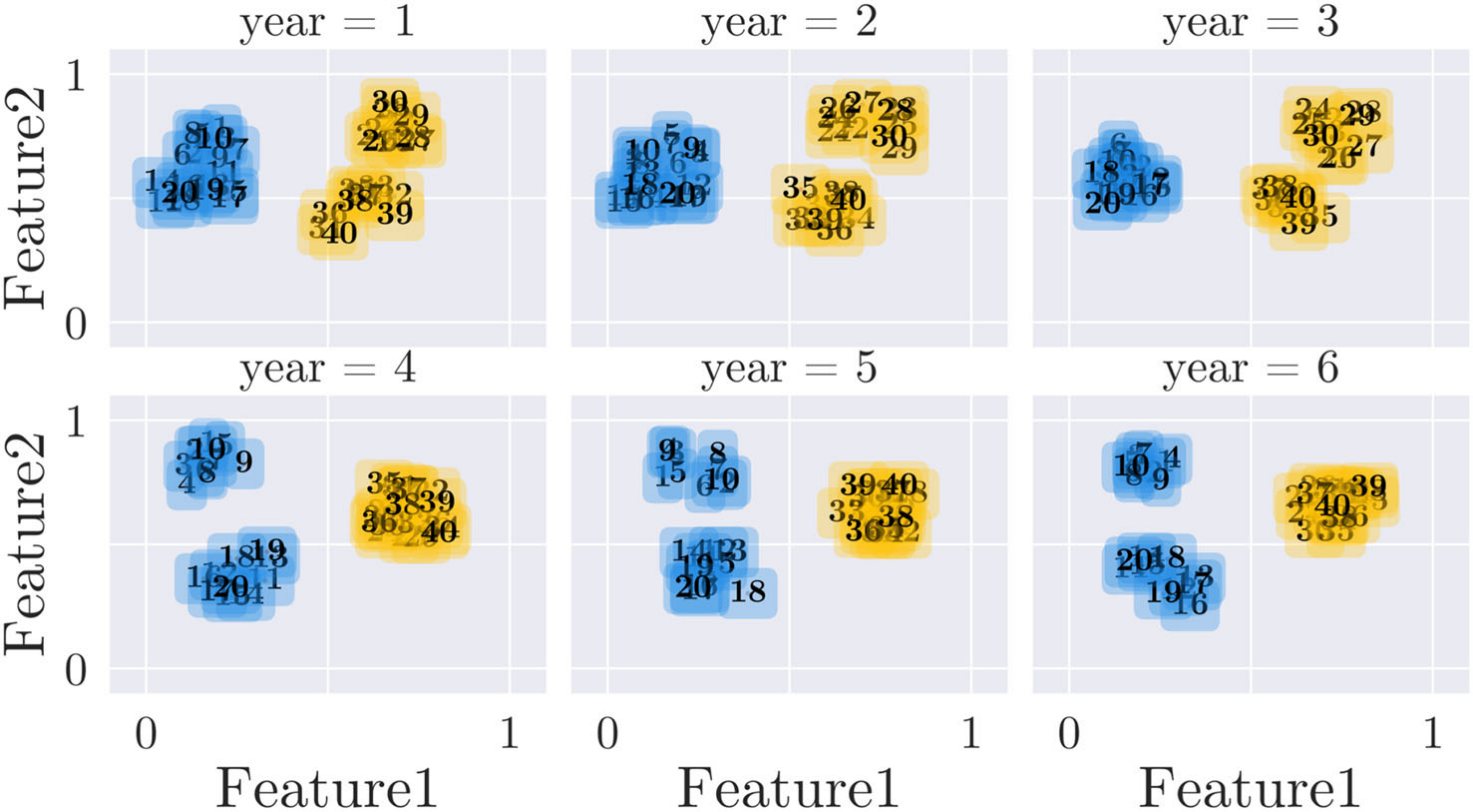
K-Means and evolutionary K-Means from [8] applied to the Generated Data Set

**Fig. 10:**
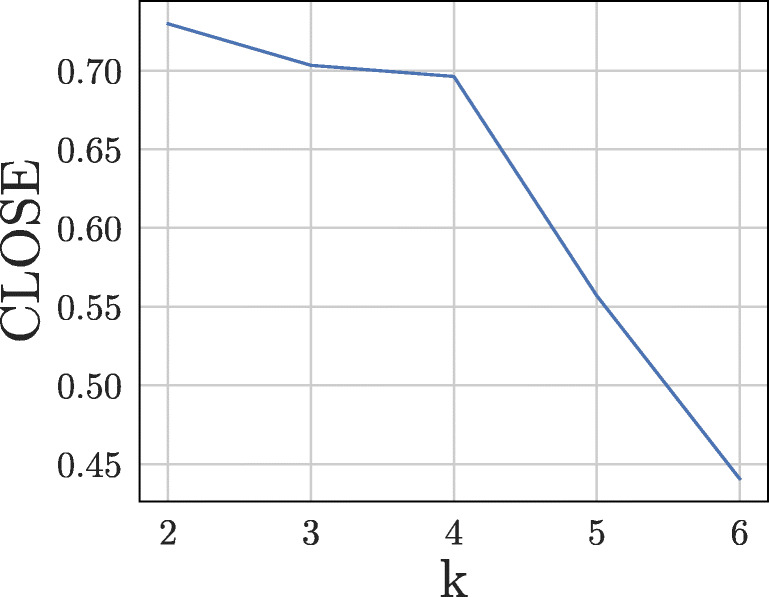
Resulting CLOSE score for standard K-Means with different *k* s

In contrast to the results with the COVID-19 data set, the clusterings of the classical K-Means and the evolutionary K-Means [8] are identical. The result can be seen in Fig. 9. This is mainly due to the nature of the generated data set. As mentioned earlier, the generated data set actually contains four clusters at each time point, two of which split off from each other and merge in *t*_4_ respectively. Although intuitively one would identify three clusters at each time point, both algorithms identified only two clusters each. This result shows that both methods recognise that categorisation into three clusters would lead to more changes within the clusters and thus to less cluster stability. The only clustering that could compete with this clustering in terms of stability would be one in which all four original clusters were identified. However, this result is not achievable due to the partitioning property of K-Means. The relatively large distance between the clusters does not prevent the evolutionary algorithm from recognising only two clusters. This can be explained by the high influence of history costs. In this case, we have set the weighting of the change parameter to 0.5 again; it can be assumed that the result will be different with a lower weight. In fact, a much lower weight leads to the detection of three clusters. We identified the *k* that leads to a clustering with the highest stability for both approaches with CLOSE (*k* = 2). Here we examined all *k* in the interval [2,6]. In Fig. 10 one can see the development of the CLOSE score as a function of the chosen *k* for the classical K-Means. In this data set, the highest CLOSE score is reached at *k* = 2. Higher *k* s lead to lower CLOSE scores. Figure 9 gives the impression that three clusters would be more intuitive at any point in time, but the problem is that such a setup would lead to more data points changing their cluster peers over time. This circumstance then leads to less stability of the individual time series, clusters and thus the entire clustering. More clusters lead to distributions in which objects have even more changing cluster peers. It should be noted that in a scenario with more clusters, quality increases but stability decreases. Together with the stability, the pre-factor then has a higher influence than the quality.

### Fuzzy C-means

**Fig. 11:**
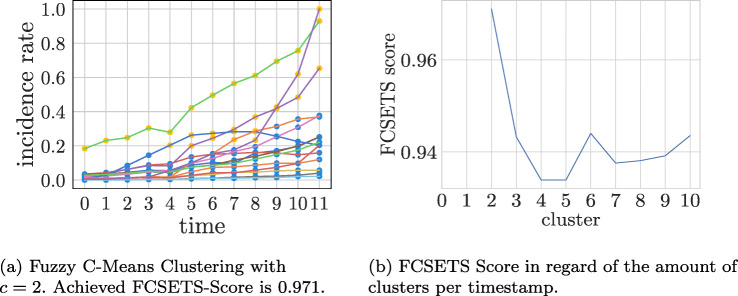
Fuzzy C-Means applied to the COVID-19 data set

In this section we discuss the results of FCSETS on the COVID-19 data set. The clusterings evaluated here were created using fuzzy C-Means, a fuzzy variant of K-Means. Figure 11b) shows the development of the FCSETS score as a function of the number of clusters.

It is noticeable that the FCSETS scores achieved are significantly higher than the CLOSE scores. This is mainly due to the fact that there is no function for evaluating the cluster quality. While the highest CLOSE score was achieved with four clusters, the highest FCSETS score was reached with two clusters (0.941). The fact that both methods evaluate different numbers of clusters with the best score is expected due to the different approaches of the underlying clustering algorithms. This also means that other clustering algorithms could achieve better or worse results in the crisp but also fuzzy case. The decisive factor for the evaluation of an over-time clustering in the fuzzy case is the change in the degrees of membership over time. Fuzzy C-Means achieves the smallest change in these with two clusters per timestamp, which also reflects the most stable result in this case.

The main reason for this is the rate of change of membership degrees from one time point to another. In the case of the COVID-19 data set, a higher number of clusters provides a higher rate of change, so that the cluster membership is less stable over time. This is especially the case when the movement of objects within clusters is high. However, usually the movement has only little influence on the highest degree of membership of an object to a cluster, but the other degrees of membership change strongly. In the case of the COVID-19 data set, this change is strongest with five clusters per timestamp. In Fig. 11a) we have visualised the clustering with the highest FCSETS score. We have assigned the objects to the cluster to which they have the highest membership degree.

### Outlier detection

In this part of the paper, we present a qualitative analysis of the presented outlier detection and its variants. In particular, we address the effects of the different proportions and weightings chosen and illustrate this using the COVID-19 data set and the generated data set. In all the analyses presented, to identify the most stable clustering, we applied CLOSE to determine the parameters.

#### COVID-19 data set

**Fig. 12:**
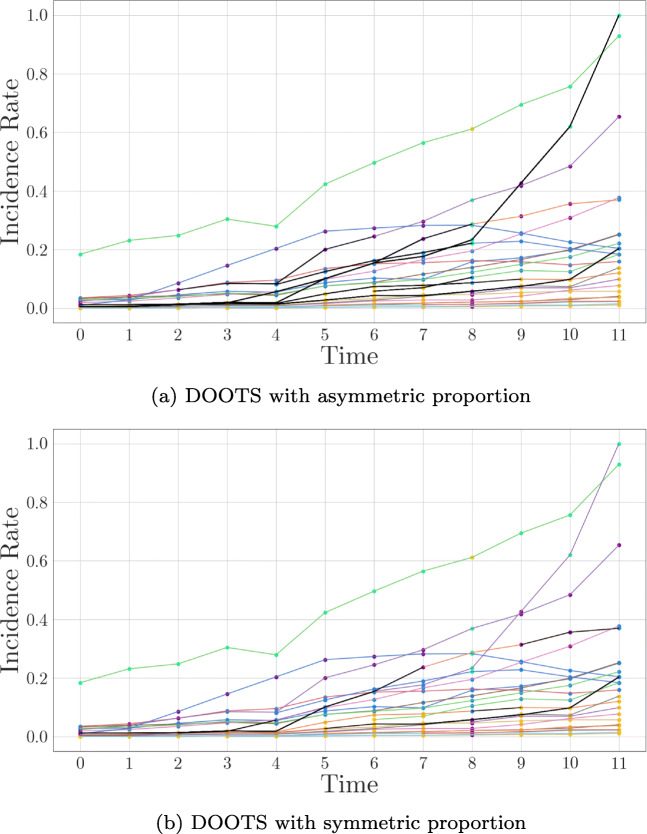
Detected outliers on the COVID-19 data set with *τ* = 0.6. black lines represent outliers. Clustering identified with CLOSE (K-Means, k = 4)

In this section we compare the effect of asymmetric proportion and symmetric (jaccard) proportion on outlier detection. For this purpose we use the one-dimensional COVID-19 data set because it is particularly suitable for illustration. We clustered the data with K-Means, identifying the most stable clustering (*k* = 4) with CLOSE. In Fig. 12 we can see the results obtained. The black graphs correspond to the outliers found. At first glance, it is immediately apparent that the outlier detection method with the symmetric jaccard proportion detects significantly fewer outliers than its asymmetric counterpart. This is due to the different evaluation of merged clusters. While merges of clusters have no influence with the asymmetrical proportion, the symmetrical jaccard proportion evaluates them negatively. This has a direct impact on the *subsequence*_*score* s, in the sense that they all become smaller in our example. This is reflected accordingly in the *best*_*score*, which corresponds to the maximum *subsequence*_*score* of a cluster. Overall smaller *subsequence*_*scores* also lead to smaller *outlier*_*scores*, because the difference between the *best*_*score* and the individual *subsequence*_*score* s also becomes smaller. With constant *τ*, as in this example, this leads to a smaller outlier detection rate. So in the case of the COVID-19 data set, we would prefer the outlie detection method with asymmetric proportion. The one-dimensional example also illustrates the type of outliers detected. In particular, we notice a time series that was detected as a whole by the system and has the highest incidence rate at the end. This time series is the incidence value of Luxembourg. There, on 31 May 2020, the highest incidence value of the European countries we looked at was reported. The high number of changes in the cluster environment is particularly striking. The first change occurs from week four to week five, followed by the change in week seven to week eight and finally the change from week nine to week ten. The constant change of cluster members leads to a relatively small *subsequence*_*score*, which then shows a high difference to the *best*_*score* s of the individual clusters.

Another rather inconspicuous time series detected by outlier detection has an incidence rate just above 0.2 at the last time point. This time series reflects the development of the pandemic in Romania. It is detected mainly because it completely changes its cluster members twice. Firstly, the incidence rate in Romania at time one does not develop like that of its cluster members at time zero: In contrast to Romania’s cluster members at time zero, the incidence rate in Romania does not continue to rise but remains at about the same level. The other change occurs from time ten to time eleven: Here, Romania’s incidence rate jumps within one week, so that it is now in a cluster with countries of a higher infection level.

In this example, the difference between the two applied proportions is not only that the asymmetric proportion detects more outliers. The jaccard proportion also detects other outliers. Exemplary for this is the sequence of the top orange-colored time series. This is detected by the outlier detection with jaccard proportion, since a merge of clusters takes place in the last time point and this is penalised by the symmetrical proportion. This is not the case with the asymmetric proportion, the merge has no effect on the *subsequence*_*score* of the time series.

Overall, relatively many outliers are found in this example. This is mainly due to the choice of the parameter *τ* and the relatively over-time stable composition of the time series. The clustering has many time series that remain in a cluster over time with comparatively many time series. This leads to high *subsequence*_*score* s and thus to high *best*_*score* s. Time series that change their cluster members only once have a comparatively low *subsequence*_*score*, which also leads directly to classification as outliers due to the selected *τ*. This example also shows how to deal with missing data: A time series only begins in the sixth week, its sequence from week six to week eight is recognised as an outlier. On the one hand, this can be explained by the change in the cluster composition from week seven to week eight and, on the other hand, by the shortness of the time series.

### ElectricityLoadDiagrams20112014 data set

**Fig. 13:**
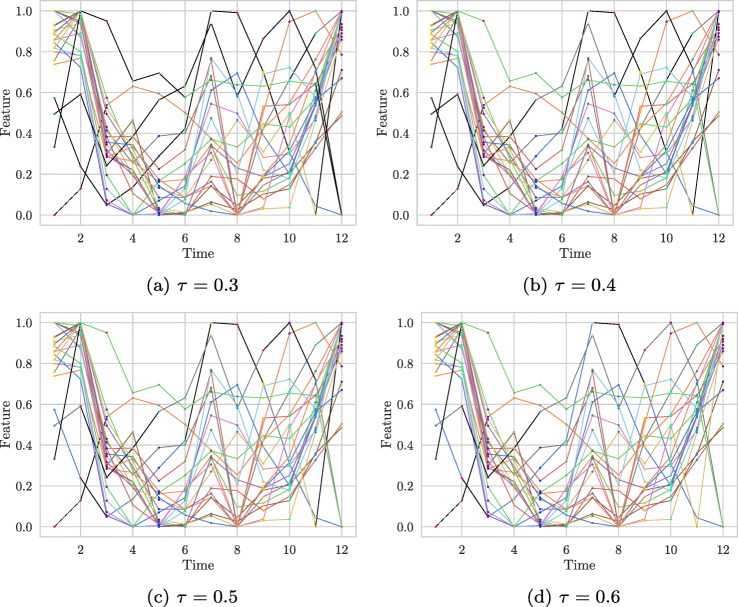
DOOTS applied to the ElectricityLoadDiagrams20112014 data set. Outliers are marked with black lines. *Intuitive outliers* are marked with black dashed lines

We clustered the one-dimensional data set with DBSCAN and determined the best parameters with CLOSE. The highest CLOSE score of 0.323 was achieved with *𝜖* = 0.11 and *minpts* = 2. The aim of this experiment is to show the influence of the outlier parameter *τ* of the DOOTS algorithm, therefore we applied four different *τ* to the clustered data set. The low CLOSE score is due to the large differences in the consumption data, which make clustering fundamentally difficult. As shown in Fig. 13, one or two clusters were identified at each time point. As expected, electricity consumption is highest in the winter months for most time series. It is also interesting to note that most time series show a local maximum in July (time 7). The expected outliers are the time series that either move completely independently of other time series or those that move with other time series at some points but then diverge from them. Figure 13a shows the result with *τ* = 0.3 and has the highest number of outliers in the four figures in Fig. 13. For example, an interesting outlier in this figure is the top time series at time 3, which was detected as an outlier from time 2 to time 6. This has to do with the comparatively low subsequence score of the time series at these times. The subsequence score of the time series is obviously even significantly worse than the subsequence score of the time series in the same cluster at time point 4. This other time series was classified as an *intuitive outlier* in the first two time points, so that these time points fall out of the calculation for the subsequence score. The top time series at time 3 is no longer recognised as an outlier from *tau* = 0.4. The difference in the subsequence score to the time series with which it was clustered is therefore obviously greater than or equal to 0.3 but less than 0.4. The differently detected outliers with the different *tau* differ not only in number but also in length. For example, the top sequence at time 7 with *τ* = 0.4 is detected as an outlier from time 6 to time 10, while the same time series with *τ* = 0.6 is only detected as an outlier from time 6 to time 9.

This indicates that the subsequence score diverges from the other time series even before the actual detection. The *τ* thus determines, among other things, how early an outlier is already classified as such.

#### Generated data set

**Fig. 14:**
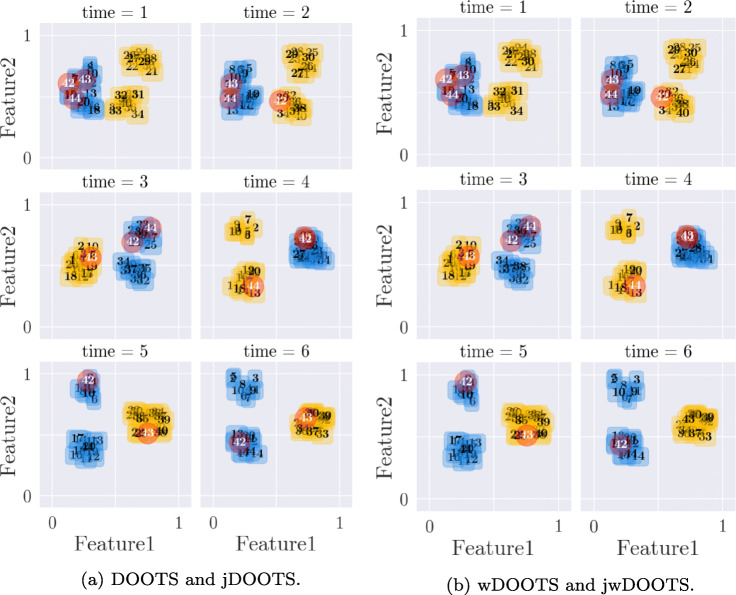
Detected outliers on the generated data set with *τ* = 0.5. Red data points represent outliers

For the evaluation of DOOTS on the generated data set, the clustering setting achieving the best CLOSE score was chosen as underlying clustering. Therefore, K-Means with *k* = 4 was used. Figure 14 shows the detected outlier sequences on the bivariate data set. All four proposed derivatives of our algorithm have been tested: the *original* method (DOOTS), the one using the *jaccard* index in the proportion calculation (jDOOTS), the one using a *weighting* in the subsequence score (wDOOTS) and the method combining the *weighting* and the *jaccard* index (jwDOOTS).

As can be seen, both approaches using the weighting function got the same results (Fig. 14b). The same applies to the remaining two (Fig. 14a). Both results are very similar to each other, as they differ only at one timestamp and that is the last one. Each method detects all three outlier sequences (42, 43, 44) in the first four timestamps. At time 5, all approaches are in agreement that there are only two outliers: 42 and 43. But at the last timestamp the weighted methods mark only one sequence (42) as an outlier, while the other ones additionally detect the time series 43.

In the first three timestamps, the detection of 42 and 44 are intuitive as they have transitions between the blue (left) and the yellow (right) cluster. In order to understand, why the sequence 43 has been marked as outlier, however, the fourth timestamp has to be inspected. Here, the sequence moves from the blue to the yellow cluster. Since both clusters have many members, which move stably over time, one transition can suffice for a high outlier score. Since all pairs of timestamps (*t*_*i*_,*t*_*j*_) with *i* < *j* are considered in the calculation of the subsequence score of a sequence ending at *t*_*j*_, the stable behavior of 43 from time 4 to 6 decreases the subsequence score even more after the transition. The subsequences $T_{t_1,t_3,43}$ and $T_{t_4,t_6,43}$ get high scores, since those sequences have a perfectly stable behavior. In context of the whole sequence, however, the score is very low, as half of the time there are completely different cluster members near the sequence than the rest of the time span.

In contrast to that, the sequence 44 is not marked as an outlier in the last two timestamps although it has more transitions than 43. This can be explained by the fact, that for 5 of 6 timestamps it is assigned to the blue cluster. Therefore, only the transition to the yellow cluster at timestamp 3 is suspicious. As already explained before, this transition has a high impact on the outlier score caused by the high stability of the other cluster members.

The impact of the weighting function gets clear considering the sequence 43 in the last timestamp. While it is marked as an outlier in Fig. 14a), it does not get a high outlier score in the weighted approaches (b). Since the impact of the timestamps of the nearer past is weighted higher than this of the more distant one, the stability of 43 after its transition at timestamp 4 is rewarded. Due to the stable behavior in the later timestamps the negative influence of the transition is compensated.

## Conclusion & future work

In this paper, we gave a short overview for a tool set specialised on time series analysis for databases containing multiple multivariate time series. The presented over-time stability evaluation measures CLOSE and FCSETS are useful tools for the evaluation of fuzzy and hard clusterings retrieved by evolutionary or time-independent clustering algorithms. With the help of CLOSE/FCSETS fitting hyperparameters for a stable over-time clustering using common clustering algorithms like K-Means [38] or DBSCAN [16] and evolutionary clustering algorithms such as evolutionary K-Means [8] may be determined. The considered definition of over-time stability varies slightly from the one usually used e.g. in evolutionary clustering. Instead of rating the actual movement of a sequence or cluster in the feature space, the behavior of a sequence is analysed in comparison to its peers. The stability of a cluster is thereby driven by its members. Also, not only the immediately preceding timestamp is considered, but the whole history of a sequence. Based on CLOSE various further TS analyses may be derived. In this paper, we e.g. propounded an outlier detection algorithm, called DOOTS, for the detection of transition-based outliers, which were firstly introduced in [50]. Two application-based modifications regarding the calculation of the *proportion* and the *subsequence_score* are shown, which may be applied to DOOTS as well as CLOSE. Because of that, the presented methods are quite flexible which makes them applicable to a broad field of applications.

The discussed experiments showed, that all depicted methods fulfill the desired intention. With the help of CLOSE common clustering algorithms are able to compete against evolutionary clusterings regarding a stable over-time clustering. In addition, CLOSE can be helpful when using evolutionary clustering algorithms in order to find the optimal parameter setting. Due to the variable components in CLOSE, such as the quality measure, it can be adapted for different types of clusterings, e.g. partition-based and density-based clusterings, in order to ensure a high quality apart from the over-time stability. This has been shown by experiments on different artificial and real-world data sets, and various clustering algorithms. Also, the influence of different parameter settings on the CLOSE score may be discovered by plotting a diagram similar to our experiment, which allows a further analysis of the underlying data.

With an underlying over-time stable clustering, the outlier detection algorithm can be applied. Our experiments showed that the desired outlier type has been detected. On lucid data sets with one or two features, those outliers may be easy to recognize with the human eye, but considering multivariate time series with higher dimension, the problem gets quite complex. Therefore, an outlier detection algorithm addressing this type of outliers might be helpful.

Apart from the presented ones, further methods based on CLOSE may be developed, e.g. an over-time clustering algorithm [29] or the prediction of the further course of sequences or clusters. Similar subsequences and patterns may already be identified by investigating the resulting clusters. Of course, an automation might easily be implemented. Since CLOSE only considers the past history of a sequence, it also may be adapted for streaming data. This could e.g. be realised by using a sliding window, which also could be included in order to speed up the run time. Generally, future work might focus on run time optimization leading to the usage of CLOSE becoming more attractive.

## Data Availability

Code online available: github.com/tatusch/ots-eval

## Footnotes

1 https://www.bsg.ox.ac.uk/research/research-projects/coronavirus-government-response-tracker

2 https://github.com/tatusch/ots-eval

3 https://github.com/CSSEGISandData/COVID-19

4 https://www.theglobaleconomy.com

## Availability of data and material

Generated data sets online available: github.com/tatusch/ots-eval
